# Interim safety analysis of the first-in-human clinical trial of the Versius surgical system, a new robot-assisted device for use in minimal access surgery

**DOI:** 10.1007/s00464-020-08014-4

**Published:** 2020-09-28

**Authors:** Dhananjay Kelkar, Mahindra A. Borse, Girish P. Godbole, Utkrant Kurlekar, Mark Slack

**Affiliations:** 1 Deenanath Mangeshkar Hospital and Research Center, Pune, Maharashtra India; 2 CMR Surgical Ltd, 1 Evolution Business Park, Milton Road, Cambridge, CB24 9NG UK

**Keywords:** Clinical trial, Minimally invasive surgical procedure, Robotic surgical procedures, Gynaecologic surgical procedures, Hysterectomy, Cholecystectomy

## Abstract

**Objective:**

The aim of this study was to provide an interim safety analysis of the first 30 surgical procedures performed using the Versius Surgical System.

**Background:**

Robot-assisted laparoscopy has been developed to overcome some of the important limitations of conventional laparoscopy. The new system is currently undergoing a first-in-human prospective clinical trial to confirm the safety and effectiveness of the device when performing minimal access surgery (MAS).

**Methods:**

Procedures were performed using Versius by a lead surgeon supported by an operating room (OR) team. Male or female patients aged between 18 and 65 years old and requiring elective minor or intermediate gynaecological or general surgical procedures were enrolled. The primary endpoint was the rate of unplanned conversion of procedures to other MAS or open surgery.

**Results:**

The procedures included nine cholecystectomies, six robot-assisted total laparoscopic hysterectomies, four appendectomies, five diagnostic laparoscopy cases, two oophorectomies, two fallopian tube recanalisation procedures, an ovarian cystectomy and a salpingo-oophorectomy procedure. All procedures were completed successfully without the need for conversion to MAS or open surgery. No patient returned to the OR within 24 h of surgery and readmittance rate at 30 and 90 days post-surgery was 1/30 (3.3%) and 2/30 (6.7%), respectively.

**Conclusions:**

This first-in-human interim safety analysis demonstrates that the Versius Surgical System is safe and can be used to successfully perform minor or intermediate gynaecological and general surgery procedures. The cases presented here provide evidence that the Versius clinical trial can continue to extend recruitment and begin to include major procedures, in alignment with the IDEAL-D Framework Stage 2b: Exploration.

**Electronic supplementary material:**

The online version of this article (10.1007/s00464-020-08014-4) contains supplementary material, which is available to authorised users.

Minimal access surgery (MAS) can help minimise blood loss, reduce post-operative complications and post-operative pain, shorten hospital stays and accelerate recovery times [1, 2]. However, MAS is associated with specific challenges; for example, the range of surgical movement is restricted, and the technique is associated with unfavourable ergonomics for the surgeon and bedside assistant. Surgeon competency is generally associated with a steep learning curve and longer training requirements. This has resulted in suboptimal MAS uptake and ultimately fewer patients benefitting from MAS [3–7].

Robot-assisted MAS has made progress in overcoming these challenges by providing a stable magnified three-dimensional view, tremor filtration, motion scaling and articulated or wristed instruments with greater degrees of movement, allowing for precise tissue dissection and suturing [8–12]. As a result, robotic surgery extends the feasibility of MAS to patients with more complex pathology and higher body mass indices (BMI), allowing a wider range of patients to benefit from the advantages of MAS [11, 13, 14]. Furthermore, surgical robots can be designed to improve the experience of the surgeon and surgical team, enhancing both career longevity and surgical outcomes [15].

The Versius Surgical System is a new tele-operated robotic surgical system (CMR Surgical, Cambridge, UK) designed to assist surgeons in performing MAS. The surgical system was developed using feedback from surgeons and surgical teams, aiming to improve both end-user experience and surgical outcomes [15]. Specifically, Versius has been designed to mimic the articulation of the human arm, with the wristed instrument tip providing seven degrees of freedom inside the patient, allowing greater surgical access compared with standard laparoscopic surgery (Fig. 1). The open console design allows surgeons to sit or stand and enables easier communication between the surgeon and the team, facilitating both training and teaching. The surgeon interacts with the system through hand controls—which mirror the general design of video gaming controllers—and visual feedback on the surgeon console. The console’s head-up display (HUD) relays the three‑dimensional video from the endoscopic camera together with a display overlay. Each instrument and visualisation arm is attached to its own wheeled cart to form a compact and mobile bedside unit (BSU), providing maximum flexibility in the operating room (OR) [15].

**Fig. 1:**
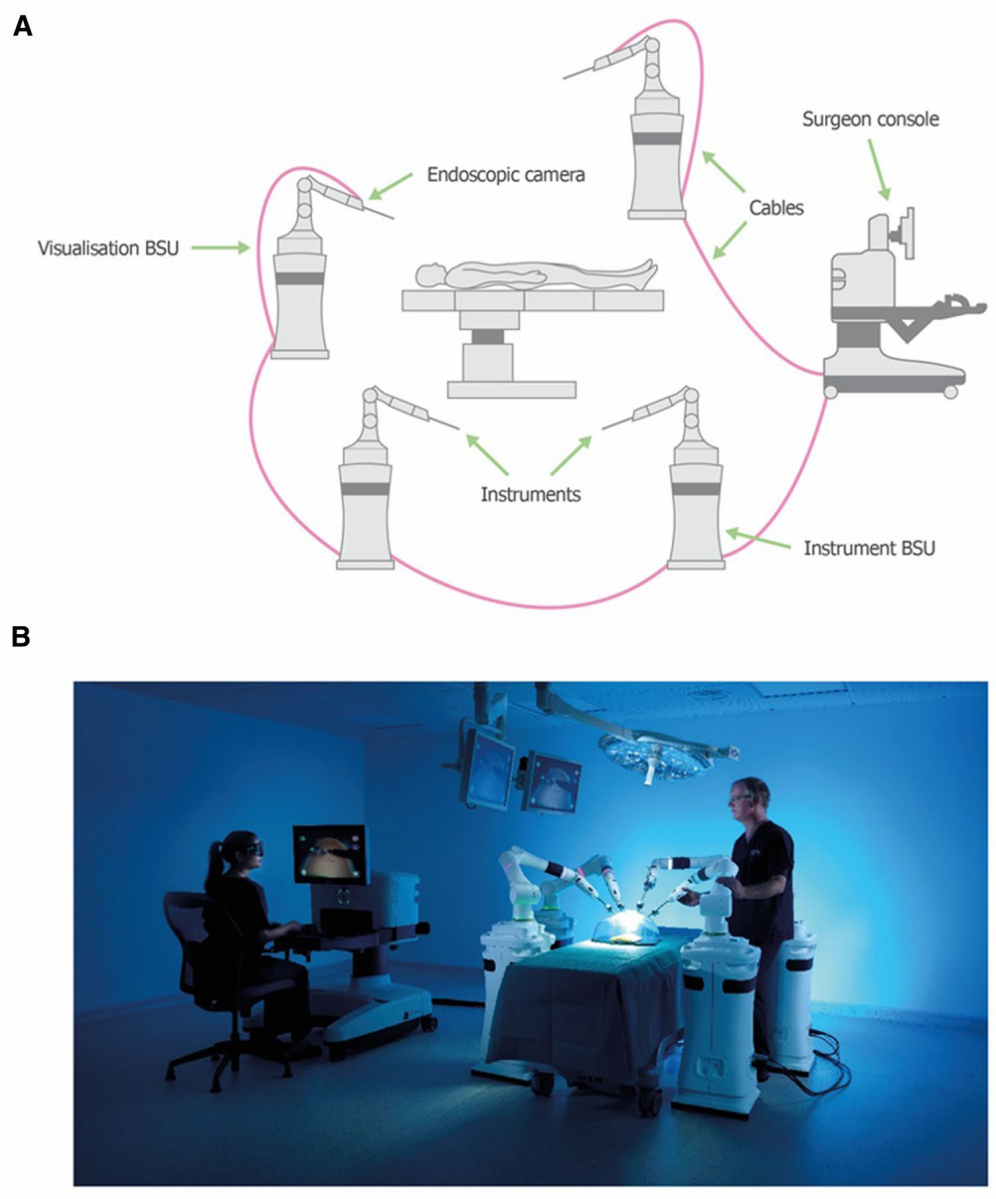
Overview of the Versius Surgical System. Adapted from Haig et al. [16]. **A** Schematic representation of the setup of Versius. **B** An image of the real-world setup of Versius. BSU: bedside unit

The operational safety and ease of use of the system was validated previously in a human cadaver study [16]. Likewise, the feasibility of Versius for transanal and mesorectal excision has also been evaluated [17]. Additional preclinical studies demonstrated that Versius can be used to successfully complete a range of gynaecological, urological, renal and general surgical procedures in both cadavers and live porcine studies [18–20].

These preclinical studies have fulfilled Stage 0 of the IDEAL-D (Idea, Development, Exploration, Assessment, Long-term follow-up) Framework—a set of recommendations for improving the evidence base from research at each stage of surgical innovation [21, 22]. The goal of the initial preclinical and Idea stages (Stage 0 and 1) is proof of concept—this was demonstrated through usability studies and procedure-specific assessment in cadavers and porcine models. [15, 16, 18–20]. These studies provided an opportunity to optimise the operational set up of the robot by confirming optimal BSU positions, port placings and procedural steps. Difficulties identified at this stage were corrected prior to proceeding to live clinical work.

The study reported herein aims to complete Stage 2a: Development. This stage seeks to refine the technique and report prospective development studies to demonstrate safety and success in performing the procedure in live humans [21, 22]. Versius is currently undergoing a prospective clinical trial to confirm the safety and effectiveness of the device when performing MAS procedures. This study reports the first-in-human use of Versius and aimed to (1) provide an initial safety analysis of 30 patients requiring minor or intermediate gynaecological or general surgery procedures, and (2) demonstrate system safety to support larger clinical trials (in line with the IDEAL-D Framework) [1, 22].

## Methods

### Ethical board review statement

Procedures were completed at the Deenanath Mangeshkar Hospital and Research Center, Erandwane, Pune, Maharashtra 411004, India, between 6 March and 2 April 2019. This study was reviewed and approved by the Institutional Ethics Committee, Deenanath Mangeshkar Hospital & Research Center. Approval for the study was received on 23 February 2019. This study has been registered on the Indian Clinical Trials Register (CTRI/2019/02/017872). All study activities were performed in compliance with ICH Good Clinical Practice Schedule Y, Indian Council of Medical Research and ISO14511 standards.

### Study population

Potential patients were identified from Deenanath Mangeshkar Hospital surgical lists and approached directly by their surgeon or clinical team between the 4 March and 2 April 2019. Male or female patients aged between 18 and 65 years old and deemed suitable for at least one surgical procedure using Versius were enrolled. After being provided with relevant study information, patients provided written and audio-visual consent. Patients were again asked for confirmation of consent before the start of surgery. In keeping with the requirements of the Institutional Ethics Committee, Deenanath Mangeshkar Hospital & Research Center and in addition to the signed written consent, the consenting process was filmed and retained.

Patients requiring the following elective minor or intermediate gynaecological or general surgical procedures were eligible for the study: salpingectomy (unilateral or bilateral), salpingo-oophorectomy, oophorectomy (unilateral or bilateral), ovarian cystectomy for benign disease, robot-assisted total laparoscopic hysterectomy (RALH), appendectomy, cholecystectomy or diagnostic laparoscopic procedures.

All patients were suitable for MAS and baseline demographics were recorded at the time of screening. For a full list of inclusion/exclusion criteria see Supplemental Table 1.

**Table 1:** Summary of patient clinical data

Case/procedure	Primary diagnosis/indication	Gender	Age (years)	BMI (kg/m^2^)	Previous surgery?* Within the last year?
1. Diagnostic laparoscopy	Primary infertility	Female	34	18.5	N
2. Ovarian cystectomy and endometriosis (Grade IV)^†^	Ovarian cyst	Female	23	22.4	N
3. Laparoscopic oophorectomy and endometriosis (Grade III)^†^	Left ovarian dermoid cyst	Female	38	23.0	N
4. Hysteroscopy, laparoscopy cannulation, polycystic ovarian drilling	Secondary infertility with bilateral tubal block	Female	30	29.9	N
5. Diagnostic laparoscopy sos cannulation	Primary infertility	Female	28	23.2	N
6. Secondary infertility with left tubal block for recanalisation	Secondary infertility and left tubal block	Female	37	20.2	Y; not within the last year
7. Diagnostic laparohysteroscopy	Secondary infertility for laparoscopy	Female	30	28.6	Y; not within the last year
8. Diagnostic laparoscopy	Primary infertility	Female	30	29.0	N
9. Appendectomy	Appendicitis	Male	31	15.5	N
10. Bilateral oophorectomy	Breast cancer, advised oophorectomy	Female	48	18.6	N
11. Cholecystectomy	Cholelithiasis	Female	40	27.0	N
12. Appendectomy	Appendicitis	Female	34	14.0	N
13. RALH	Uterine bleeding with adenomyosis	Female	48	24.8	N
14. Diagnostic laparoscopy	Primary infertility	Female	35	24.0	N
15. RALH	Abnormal uterine bleeding	Female	48	22.6	Y; within the last year
16. RALH	Pelvic inflammatory disease	Female	28	25.9	Y; not within the last year
17. Cholecystectomy	Cholelithiasis	Female	41	33.4	N
18. Right salpingo-oophorectomy	Right ovarian complex cyst	Female	40	28.0	N
19. Cholecystectomy	Cholelithiasis	Male	53	23.8	N
20. Appendectomy	Appendicitis	Male	30	34.3	N
21. Appendectomy	Appendicitis	Female	27	19.2	N
22. Cholecystectomy	Cholelithiasis	Female	33	27.4	N
23. RALH	Adenomyosis with fundal fibroid	Female	44	29.0	N
24. Cholecystectomy	Cholelithiasis	Female	42	24.0	N
25. Cholecystectomy	Cholelithiasis	Male	38	28.3	N
26. Hysterectomy with salpingo-oophorectomy	Adenomyosis, endometriosis	Female	38	33.5	Y; not within the last year
27. Cholecystectomy	Cholelithiasis	Female	39	39.8	Y; not within the last year
28. Cholecystectomy	Pancreatitis with cholelithiasis	Male	64	22.6	N
29. Cholecystectomy	Cholelithiasis	Female	46	32.6	Y; not within the last year
30. RALH	Menorrhagia with adenomyosis with adenomyoma	Female	37	25.0	Y; not within the last year

^*^Pelvic/abdominal surgery. ^†^Endometriosis only discovered after insertion of endoscope

BMI: body mass index; N: no; RALH: robot-assisted total laparoscopic hysterectomy; Y: yes

### Study design

**Fig. 2:**
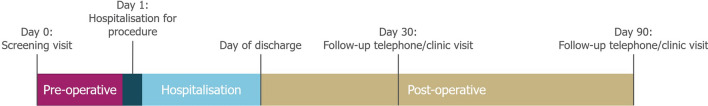
Schematic overview of the study design

Following patient screening, hospitalisation and discharge, patients had follow-up clinical visits or telephone calls at 30 and 90 days post-operation (Fig. 2). Patients were under daily post-operative surveillance while an in-patient and the next case was not initiated until the surgical team and chief medical officer were satisfied that the preceding case was a success and the patient was not experiencing any adverse effects due to suboptimal performance of Versius. Accordingly, the first 10 cases were deliberately chosen to be minor or diagnostic procedures, before attempting more complex cases.

The primary endpoint was the rate of unplanned conversion of procedures to other MAS techniques or open surgery. Secondary endpoints included intra-operative complications, complications occurring during hospital stay or within 90 days after discharge. All post-operative complications were graded according to the Clavien–Dindo classification [23]. Additional secondary endpoints included: intra‑operative blood transfusion, estimated intra‑operative blood loss, return to the OR within 24 h, return to the OR after 24 h, readmission to hospital within 30 and/or 90 days, operative time (from incision to skin closure), length of hospital stay and 90‑day mortality. BSU and port positions in relation to anatomical landmarks were also recorded.

### Surgical team and system setup

Procedures were performed by a lead surgeon supported by an OR team. The lead surgeon performed the surgical steps for the procedure from the surgeon console. The bedside assistant manipulated the robotic arms and carried out any additional manual tasks as instructed by the lead surgeon. All members of the surgical team completed and passed the validated 3.5-day Versius training programme prior to the start of the study, as per the Versius training protocol [24]. The six lead surgeons who performed the procedures were accredited, practising, high-volume gynaecological or general consultant surgeons.

**Fig. 3:**
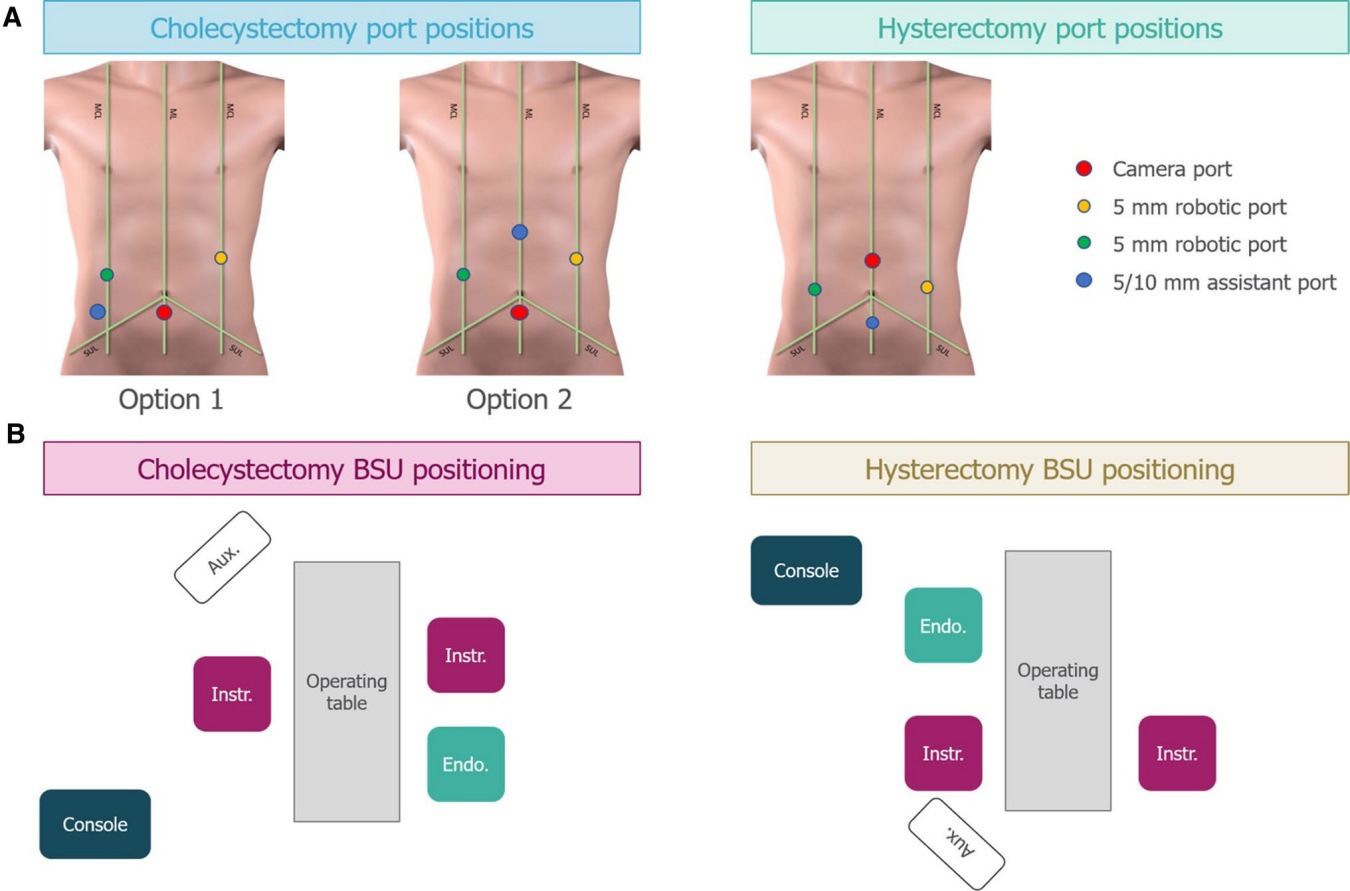
Common operative setup for cholecystectomy and hysterectomy procedures. **A** Common port positions for cholecystectomy and hysterectomy procedures with corresponding BSU positions shown below in **B**. The assistant port was for nonrobotic laparoscopic instruments. Umbilicus is where the ML crosses the SUL. Aux: auxiliary monitor; BSU: bedside unit; Console: surgeon console; Endo: endoscope; Instr: instrument; MCL: midclavicular line; ML: midline; SUL: supine-umbilical line

The port placement for cholecystectomy and RALH procedures are shown in Fig. 3a. For cholecystectomy procedures, the camera port was positioned up to 2 cm below the umbilicus on the midline, with a 5 mm robotic port on the right and left midclavicular line (MCL). Either a 5 mm or 10 mm assistant port was positioned either superior to the iliac crest (option 1) or in the epigastrium (Fig. 3a; option 2). For high BMI patients, the camera port was positioned above the umbilicus. For RALH procedures, the camera port was positioned up to 2 cm above the umbilicus on the midline, with a 5 mm robotic port on the right and left MCL, at the level of the umbilicus. A 5 mm assistant port was positioned below the umbilicus at the midline. For high BMI patients, the camera port was positioned below the umbilicus. The most frequent operational setup for cholecystectomy and RALH is represented in Fig. 3b.

## Results

### Patient demographics and procedures

Of the 30 patients included in the analysis, the majority were female (83.3% female, 16.7% male), with a median age of 37.5 years (range: 23–64 years; Table 1). Median BMI was 24.9 kg/m^2^ (range: 14.0–39.8 kg/m^2^) and all but one patient (Case 15) had not undergone pelvic or abdominal surgery within the last year.

In total, 13 general surgical procedures were performed: 9 cholecystectomies with a primary diagnosis of cholelithiasis and 4 appendectomies with a primary diagnosis of appendicitis. The majority of the gynaecology cases required RALH (six cases) or diagnostic laparoscopy (five cases); the remaining cases required oophorectomy (two cases), fallopian tube recanalisation (two cases), ovarian cystectomy (one case), or salpingo-oophorectomy (one case). The primary indication for RALH was bleeding abnormalities resistant to conservative management. A complete list of specific diagnoses and procedures is shown in Table 1.

### Patient outcomes

No procedure required conversion to conventional MAS or open surgery, all procedures were completed successfully and there were no intra-operative complications. The procedures were performed according to the procedural steps described in the preclinical studies, therefore there was no need to modify steps during this clinical phase [18, 19]. Intra-operative blood loss was estimated as negligible (< 5 mL) for 19/30 (63.3%) procedures or minimal (< 500 mL) for 11/30 (36.7%) procedures; only one case (3.3%; Case 15) required the use of blood transfusion products (required post-operatively and not related to intra-operative blood loss reported). No patient returned to the OR within or after 24 h of surgery, and readmittance rate at 30 and 90 days was 1/30 (3.3%) and 2/30 (6.7%), respectively. Both cases (Cases 24 and 25) were acute gastroenteritis of Clavien–Dindo Grade I and were not related to the surgical device. Patients were treated symptomatically with pain killers and antiemetics and made a full recovery. Median operative time was 120 min (range: 35–306 min; Table 2); extended operating times reflect the degree of caution taken by the surgical team as they gained familiarity with the system and instrumentation. Median length of hospital stay was 3 days (range: 2–10 days) and 90-day mortality was 0% with all patients completing the study.

**Table 2:** Summary of patient outcome data

Case/procedure	Operative time (mins)	Conversion?	Estimated intra-operative blood loss (mL); blood transfusion required?	Return to OR within 24 h?	Length of hospital stay (days)	Intra-and post-operative complications	Readmitted to hospital
1. Diagnostic laparoscopy	75	N	< 5; N	N	2	N	N
2. Ovarian cystectomy and endometriosis (Grade IV)^†^	165	N	< 500; N	N	2	N	N
3. Laparoscopic oophorectomy and endometriosis (Grade III)^†^	75	N	< 5; N	N	2	N	N
4. Hysteroscopy laparoscopy sos cannulation sos polycystic ovarian drilling	60	N	< 5; N	N	2	N	N
5. Diagnostic laparoscopy sos cannulation	45	N	< 5; N	N	2	N	N
6. Secondary infertility with left tubal block for recanalisation	35	N	< 5; N	N	2	N	N
7. Diagnostic laparohysteroscopy	60	N	< 5; N	N	2	N	N
8. Diagnostic laparoscopy	90	N	< 5; N	N	2	N	N
9. Appendectomy	90	N	< 5; N	N	4	N	N
10. Bilateral oophorectomy	60	N	< 500; N	N	3	N	N
11. Cholecystectomy	150	N	< 500; N	N	2	N	N
12. Appendectomy	80	N	< 5; N	N	3	N	N
13. RALH	210	N	< 5; N	N	4	N	N
14. Diagnostic laparoscopy	45	N	< 500; N	N	3	N	N
15. RALH	120	N	< 5; Y	N	7	N	N
16. RALH	120	N	< 5; N	N	3	N	N
17. Cholecystectomy	60	N	< 5; N	N	2	N	N
18. Cholecystectomy	120	N	< 5; N	N	3	N	N
19. Cholecystectomy	150	N	< 500; N	N	3	N	N
20. Appendectomy	120	N	< 5; N	N	7	N	N
21. Appendectomy	135	N	< 5; N	N	2	N	N
22. Cholecystectomy	150	N	< 5; N	N	3	N	N
23. RALH	210	N	< 5; N	N	4	N	N
24. Cholecystectomy	135	N	< 500; N	N	2	Y;PO^a^	Y;^a^ within 30 days
25. Cholecystectomy	230^b^	N	< 500; N	N	10	Y;PO^a^	Y;^a^ within 90 days
26. Hysterectomy with salpingo-oophorectomy	306^c^	N	< 500; N	N	4	N	N
27. Cholecystectomy	120	N	< 500; N	N	3	N	N
28. Cholecystectomy	275	N	< 500; N	N	7	N	N
29. Cholecystectomy	195	N	< 500; N	N	2	N	N
30. RALH	140	N	< 5; N	N	6	N	N

N: no; OR: operating room; PO: post-operative; RALH: robot-assisted laparoscopic hysterectomy; Y: yes

^a^Readmitted due to acute gastroenteritis, not related to the device

^b^Required additional time to remove extensive port site adhesions and adhesions covering Calot’s Triangle

^c^Extensive endometriosis was surgically treated before performing the hysterectomy. ^†^Endometriosis only discovered after insertion of endoscope

There were two cases (Cases 2 and 3) of Grade III–IV endometriosis, only identified on insertion of the endoscope. The operating surgeon and the chief medical officer decided to proceed, on the proviso that the cases were being performed as safely as they would be with conventional surgery. The diagnoses did, however, influence operative times; 75 min for laparoscopic oophorectomy with Grade III endometriosis (Case 3) and 165 min for ovarian cystectomy with Grade IV endometriosis (Case 2). In addition, two other cases had extended operating times, a cholecystectomy (case 25) and hysterectomy (case 26) respectively. Case 25 required additional time to remove extensive port site adhesions and adhesions covering Calot’s Triangle. While extensive endometriosis was surgically treated before performing the hysterectomy in case 26. All cases were completed successfully.

## Discussion

Overall, this first-in-human interim safety analysis demonstrates that Versius is safe and feasible for use in performing minor and intermediate gynaecological and general surgery procedures. No intra-operative complications were recorded and none of the procedures required conversion to open surgery or conventional MAS. Furthermore, estimated intra-operative blood loss was negligible for 63.3% (19/30) of cases, while the remaining cases reported minimal blood loss. There was no return to the OR within or after 24 h and most patients were discharged after 3 days; however, this ranged between 2 and 10 days. A hospital stay of longer than three days is a standard precaution taken at the study institution for all RALH and cholecystectomy cases (robotic or otherwise), due to the distance between a patient’s home and the hospital and the affordability of repeated travel. It is not related to additional post-operative complications or safety of surgery with Versius.

The versatility of the system enabled procedures to be successfully completed in a wide range of patient BMIs. Over the course of the study, only two patients (6.7%) were readmitted to hospital due to acute gastroenteritis which was not device‑related. The successful completion of the first 30 cases justifies continuation of the clinical trial.

All robot-assisted surgical devices can potentially fail during procedures and cause harm or damage to internal structures or organs [25]. Versius had first undergone rigorous preclinical testing and surgical teams had been extensively trained in the use of the system to minimise the risk in this study [15–18, 20, 24]. Moreover, device safety was continuously monitored throughout surgery and each case was considered individually. Accordingly, minor cases were selected first to allow the surgeon and OR teams to gain live-surgery experience using Versius in the least stressful environment possible. However, two cases of endometriosis, only identified on insertion of the endoscope, were beyond the case complexity intended. A decision was made to proceed as the surgeon felt confident that they were within the reach of the system. These procedures were completed in the presence of the chief medical officer, who continually monitored the safe continuation of the surgery. Both cases were completed successfully and demonstrated the ability of the system to deal with advanced dissection required for treating Grade IV endometriosis, a key umbrella and indicated procedure. In addition, several more complex procedures such as RALH and cholecystectomy were successfully performed using Versius with no intra-operative complications.

Performing a procedure with a new complex surgical device is expected to be associated with a slower time of surgery as the teams gain familiarity with the system and instrumentation (e.g. not only the surgeon but also the bedside team moving Versius during the procedure). Care was being taken to ensure patient safety, hence longer operating times were recorded early in the cohorts. As hospital teams become increasingly familiar and confident, it is anticipated that operative times will decrease. However, as safety is of paramount importance, conclusions drawn from metrics such as operative times should be moderated and not taken as an authoritative measure of patient outcome.

New users of any surgical robotic system will undergo a learning curve during the training period in which they develop the skills required to safely and effectively operate the device during surgical procedures [26, 27]. To aid effective training, all study participants completed a 3.5-day-long residential training programme, representative of commercial training. The Versius training programme was developed to include both didactic and practical, hands-on training and incorporate tasks designed to develop the motor and cognitive skills required to achieve competency in using Versius. The successful completion of all procedures undertaken demonstrates the high level of competency achieved. Additionally, completion of the more complex endometriosis cases, demonstrate that the operating surgeon (who routinely operates on advanced grades of endometriosis) was confident the system provided the same surgical ability as a conventional, straight stick system.

### Study limitations

The cases presented in this study represent the first-in-human use of Versius and, as such, place additional pressures on the lead surgeons and their surgical teams. Consequently, extreme care and caution was taken throughout the procedures and may not be entirely representative of how they would be performed on a routine basis. It is anticipated that with more experience, surgical outcomes such as operative time will decrease.

## Conclusion and future perspectives

This study shows promising first-in-human clinical trial data that support previous preclinical study results [18–20]. All 30 cases presented in this analysis were completed successfully and provide evidence supporting continued recruitment into the trial and inclusion of major surgical procedures. Continuation and expansion of this trial will ensure continued alignment with the IDEAL-D Framework and aim to demonstrate evidence of framework Stage 2b: Exploration. The goal of this stage is to build upon the technique established and expand the patient base to > 100 [21, 22]. Increasing the number of operations completed with Versius will demonstrate the safety and efficacy of the robotic system and allow for comparison with other available robotic systems.

## Disclosures

DK: None declared; MB: None declared; GG: None declared; UK: None declared; MS: Chief Medical Officer and founder of CMR Surgical.

## Electronic supplementary material

Below is the link to the electronic supplementary material.

Supplementary file 1 (DOCX 49 kb)
