# Supplementary material for: Interim safety analysis of the first-in-human clinical trial of the Versius surgical system, a new robot-assisted device for use in minimal access surgery
Source: Surg Endosc. 2020 Sep 28;35(9):5193–202. doi: 10.1007/s00464-020-08014-4 (PMC8346419; doi:10.1007/s00464-020-08014-4)
Supplement: Supplementary file 1 — (DOCX 49 kb) [file 464_2020_8014_MOESM1_ESM.docx]

SUPPLEMENTARY MATERIALS

**Supplemental Table 1**. Study inclusion and exclusion criteria

| Inclusion criteria | Exclusion criteria |
| --- | --- |
| - Male or female - Age 18–65 years - If female and of childbearing age, must not be pregnant and agree to not become pregnant for the duration of the study - Deemed suitable for at least one surgical procedure using the Versius Surgical System - Patient (or appropriate legal representative) able to provide written informed consent to participate in the study | - Patient participation in an investigational clinical study within 30 days before screening - Inability to provide informed consent - Uncontrolled hypertension (≥systolic: 180 mmHg/diastolic: 120 mmHg) and/or diabetes mellitus (blood glucose level: >200 mg/dL) - New York Heart Association Class III or IV[[1](#_ENREF_1)] - Incidence of metastases, regional and/or distant - History of chronic alcohol or drug abuse - Chronic renal failure or on dialysis - Significant medical history or immunocompromised - Subjects with any other clinically significant unstable medical disorder, life-threatening disease, or anything else in the opinion of the investigator which would contra-indicate a surgical procedure |

Supplementary Reference

1. Criteria Committee of the New York Heart Association (1994) Nomenclature and criteria for diagnosis of diseases of the heart and great vessels, Boston: Little, Brown & Co.
